# Current Status and Regulatory Aspects of Pesticides Considered to be Persistent Organic Pollutants (POPs) in Taiwan

**DOI:** 10.3390/ijerph7103615

**Published:** 2010-10-12

**Authors:** Wen-Tien Tsai

**Affiliations:** Graduate Institute of Bioresources, National Pingtung University of Science and Technology, Pingtung 912, Taiwan; E-Mail: wttsai@mail.npust.edu.tw; Tel.: +886-8-7703202; Fax: +886-8-7740134

**Keywords:** organochlorine pesticide (OCPs), persistent organic pollutants (POPs), environmental distribution, regulatory control, Taiwan

## Abstract

Organochlorine pesticides (OCPs) are capable of persisting in the environment, transporting between phase media and accumulating to high levels, implying that they could pose a risk of causing adverse effects to human health and the environment. Consequently, most OCPs are designated as persistent organic pollutants (POPs) and even as endocrine disrupting chemicals (EDCs). The objective of this paper was to review the current status of pesticide POPs in Taiwan, including aldrin, chlordane, chlordecone, DDT, dieldrin, endrin, heptachlor, hexachlorobenzene, α/β-hexachlorocyclohexanes, lindane, mirex, pentachloro-benzene, and toxaphene. The information about their environmental properties, banned use, carcinogenic toxicity and environmental levels, can be connected with the regulatory infrastructure, which has been established by the joint-venture of the central competent authorities (*i.e.*, Environmental Protection Administration, Department of Health, Council of Agriculture, and Council of Labor Affairs). The significant progress to be reported is that the residual levels of these pesticide-POPs, ranging from trace amounts to a few ppb, have declined notably in recent years.

## 1. Introduction

In the past decades, many studies have focused on the potential of a diverse number of persistent organic pollutants (POPs) to cause adverse effects to the environmental and human health [1]. These chemical substances are very stable compounds that resist photolytic, biological and chemical degradation, that thus persist in the environment with long half-lives. Recently, there has been concern about the hormone-like effects on the endocrine and reproductive systems of wildlife and humans of many POPs because of their lipophilicity and tendency to accumulate in food chains and bodies [2]. Today, these chemicals are collectively termed endocrine disrupting chemicals (EDCs), endocrine disruptors or environmental estrogens.

Since the 1960s, the risks posed by POPs, especially the organochlorine pesticides (OCPs), have become of increasing concern in international communities. These OCPs are known to be widely used as insecticides, herbicides and fungicides. It should be noted that some of the OCPs also have anti-estrogenic (androgenic) activity. Most OCPs were applied in agriculture and transported to the environment by means of runoff and infiltration. Several regional and global POP treaties and/or initiatives have been negotiated for identifying POPs and developing risk management measures to reduce the exposure of humans and the ecosystem to these toxic substances [3,4]. Among these treaties, the Stockholm Convention on POPs, which came into effect on 17 May 2004, is the most important milestone. Twelve POPs, consisting primarily of OCPs, were initially listed in the Stockholm Convention to be eliminated or their production and use restricted. The OCPs or pesticide-POPs identified in the Convention include aldrin, chlordane, DDT, dieldrin, endrin, heptachlor, hexachlorobenzene, mirex, and toxaphene. At the fourth meeting of the Conference of the Parties (COP4), the depositary communicated the adoption of the amendment to Annexes A (elimination), B (restriction) and C (unintentional production) of the Stockholm Convention, that shall enter into force on 26 August 2010. Nine new POPs, including hexachlorocyclohexane, chlordecone, lindane, and pentachlorobenzene were added to the Convention.

In Taiwan, soil contamination by OCPs occurred during the 1950s–1970s period due to the widespread use of pesticides in the agricultural sector [5]. As surveyed by Vu Duc *et al.* [6], the residual levels of OCPs (e.g., DDT and HCH) in soil samples from paddy fields, uplands and urban areas in Taiwan seemed to be higher than those in other Asian countries such as Thailand and Vietnam. It was estimated that over two million kilogram of OCPs was released into the environment annually in that period via volatility, soil erosion and agricultural runoff [7]. From the surveys on the distribution of OCPs residues in river sediments of Taiwan, it was found that residues of some OCPs, such as DDT, dieldrin and hexachlorocyclohexane (HCH), were still detectable in minor amounts in soils and sediments [5,7]. The central competent authorities, including the Council of Agriculture (COA) and the Environmental Protection Administration (EPA), have thus promulgated relevant regulations to ban or restrict toxic and persistent OCPs, and also started investigations to monitor their environmental levels since the 1980s [8]. They were banned for use under the Toxic Chemical Substances Control Act and the Pesticide Management Act. While Taiwan is currently not a party to the Stockholm Convention, it has specially drafted the “National Implementation Plan of the Stockholm Convention on Persistent Organic Pollutants” to serve as a basis for domestic implementation work. This plan was approved by the Executive Yuan on 24 April 2008. Subsequently, other relevant competent authorities, including the Department of Health (DOH), the Council of Agriculture (COA) and the Council of Labor Affairs (CLOA), have also worked together to develop regulatory frameworks to solve pesticide-POP issues.

Although the use of OCPs in Taiwan has been banned for many years, the regulatory and non-regulatory approaches for these pesticide-POPs will be expected to offer prevention strategies for other countries. In line with the public concern in recent years about the human health risks of emerging pollutants, this comprehensive paper will focus on the pesticide-POPs, including aldrin, chlordane, chlordecone, DDT, dieldrin, endrin, heptachlor, hexachlorobenzene, α/β-hexachlorocyclohexanes, lindane, mirex, pentachlorobenzene, and toxaphene. The main subjects addressed by this paper cover the following key elements:

- Properties and carcinogenity of pesticides-POPs
- Use and distribution of pesticides-POPs in Taiwan
- Regulatory system for controlling pesticides-POPs in Taiwan

## 2. Properties and Carcinogenity of Pesticide-POPs

### 2.1. Properties of Pesticide-POPs

As mentioned above, POPs have been considered to be extremely stable, highly inert and unlikely to be decomposed under e normal environmental conditions. It means that the environmental fate of these pesticide-POPs if released or emitted only involves transportation from a phase to another without changing their identities until equilibrium is approached. Therefore, the properties regarding the environmental distribution among the air, water, and solid phases are very important in understanding their movement between media and evaluating their behavior within a single medium. These environmental properties commonly include octanol-water partition coefficient (*K_ow_*), water solubility (S_w_), Henry’s law constant (H), and vapor pressure (V_p_). Table 1 summarized the identities and environmental properties of these pesticide-POPs, which were mainly compiled from available monographs or books [9,10]. From the molecular structure point of view, these pesticide-POPs comprise C=C (aromatic), C-H, and C-Cl chemical bonds, with lesser numbers of C-C and C-O bonds. This means that the compounds tend to have low polarity, resulting in being lipophilic with high *K_ow_* values and having a low solubility in water. As seen in Table 1, it is obvious that these pesticide-POPs, with high log *K*_ow_ values ranging from 3.6 to 6.5, are hydrophobic compounds and have low solubilities in water which lie between < 10^−6^ mg/L for endrine to 7.6 mg/L for chlordecone. Therefore, they partition favorably to organic matter and have low mobility, limiting their capacities for spreading in the aqueous phase of soil and sediment [11].

Interface transfer between the gas phase (*i.e.*, atmospheric air) and water bodies is one of the fate processes affecting the transport of many chemical compounds in the environment because the vaporization from aqueous solutions is an important transport pathway from water to air. In this regard, Henry’s law is used to describe the partition of a vapor between two different phases, such as water and air, under equilibrium conditions. Therefore, the Henry’s law constant (H) is usually defined as the ratio of a chemical’s concentration in air to its concentration in water at equilibrium. From the data on the Henry’s law constant (H) and vapor pressure (V_p_) in Table 1, it has been suggested that these pesticide-POPs are semi-volatile with extraordinarily low vapor pressure, but tend to diffuse from water phase (*i.e.*, so-called fugacity, or escaping tendency) to the air phase due to their low solubilities in water. Although human exposure to OCPs can also occur via inhalation or by dermal absorption, ingestion is the major route of exposure in view of the health risks.

### 2.2. Carcinogenity of Pesticide-POPs

The pesticide-POPs under discussion consist of aromatic and/or alicyclic rings, and also contain carbon-halogen bonds to a different degree, suggesting that these chemicals are stable in the environment and should not be acutely toxic to living organisms. However, even when they are present in very low concentration, these POPs may pose high toxicity for their long-term and chronic effects because they are characterized as highly lipid soluble and thus bioaccumulate to a significant degree. It should be noted that these compounds are xenobiotic chemicals that may induce the hormone-like effects on the endocrine systems of wildlife and humans. The endocrine and reproductive effects of these pesticides are believed to mimic or disrupt the effect of endogenous estrogens. In the Strategic Programs on Environmental Endocrine Disruptors ’98 (SPEED ’98) by the Ministry of the Environmental Government of Japan, the pesticide-POPs except for pentachlorobenzene have been incorporated into the endocrine disrupting chemicals (EDCs) class, as shown in Table 2.

Regarding the chronic toxicity, carcinogenicity is the most important concern as compared to mutagenicity, tetragenicity, neurotoxicity and reproductive toxicity. As summarized in Table 2, the United States National Toxicology Program (USNTP) has listed chlordecone, DDT, hexachlorobenzene, hexachlorocyclohexane, lindane, mirex, and toxaphene as “reasonably anticipated to be a human carcinogen” based on sufficient evidence of carcinogenicity in experimental animals. To be in consistent with USNTP, the International Agency for Research on Cancer (IARC) has classified these pesticide-POPs and other POPs (*i.e.*, chlordane and heptachlor) as “possibly carcinogenic to humans” (group 2B). By contrast, aldrin, dieldrin and endrin are considered as Group 3 (“not classifiable as to carcinogenicity to humans”). On the other hand, the carcinogenic toxicities of these pesticide-POPs are relatively less than those of polychlorinated dibenzo-*p*-dioxins (PCDDs), polychlorinated dibenzofurans (PCDFs) and polychlorinated biphenyls (PCBs), because these polychlorinated POPs are generally expressed as their toxic equivalent factors (TEF) relative to the toxicity of 2,3,7,8-tetrachlorodibenzo-*p*-dioxin (2,3,7,8-TCDD) [12]. It is well known that 2,3,7,8-TCDD is regarded as the most toxic substance among PCDDs, PCDFs and PCBs because it has been classified as Group 1 (IARC) and known to be a human carcinogen (USNTP).

## 3. Uses and Distribution of Pesticide-POPs in Taiwan

In line with the international treaties and/or efforts since the early 1970s, the Taiwanese government has promulgated relevant regulations for banning the production, import, sale, and use of pesticide-POPs, which were widely used as insecticide. Table 3 lists the banning or restriction dates of pesticide-POPs under the authorization of the environmental laws, including the Pesticide Management Act, the Toxic Chemical Substance Control Act and the Environmental Agents Control Act.

It is seen that some pesticide-POPs (*i.e.*, chlordane, chlordecone, heptachlor, mirex and pentachlorobenzene) were never registered in Taiwan. As a consequence, the source inventories of pesticide-POPs in Taiwan were not fully developed because their use/consumption activities were insufficient to identify and/or quantify the emissions from all sources.

Basically, pesticide-POPs are not produced naturally, so their presence in the environment must be in consequence of anthropogenic activities. Inevitably, the traces of these chemicals were directly released to receiving water body and the atmosphere via permitted discharges at its manufacturing facilities (emission sources), or could be indirectly and directly emitted to the environment during the application and handling of these OCPs. From the released amount, the environmental properties and biodegradability for pesticide-POPs, we can see that their distributions in the environment could be detected as trace concentrations in soils and sediments. The central competent authorities in Taiwan, including the Council of Agriculture (COA) and the Environmental Protection Administration (EPA), have conducted monitoring surveys on soil and river bottom sediment for common pesticide-POPs. The monitoring results in the environmental media were briefly summarized as follows:

1. Since the early 1980s, the central government-level agency (*i.e.*, COA) began monitoring the residual concentrations of common OCPs in agricultural soils every 10 years. The survey data in 2004 showed that the majority of monitoring concentrations varied in the range from 0.19 to 340 ng/g-dw [8]. Analyzing the monitoring data in the soil samples, it also indicated a declining trend over the past decades.
2. The surveys on bottom sediments from principal rivers by EPA were conducted from 2002 to 2006 [8], showing that the majority of monitoring data for common OCPs (*i.e.*, aldrin, chlordane, DDT, dieldrin, endrin, heptachlor, hexachlorobenzene, and lindane) and their metabolites were less than detectable limits, and have been diminishing gradually.
3. One study reported the environmental level of OCPs in river sediments (40 samples) collected from Oct. 1997 to Apr. 1998 [7]. The concentrations of OCPs in sediments were in the ranges of 0.57–14.1 ng/g-dw for HCH, 0.05–0.15 ng/g-dw for aldrin, 0.12–5.8 ng/g-dw for dieldrin, 0.22–0.64 ng/g-dw for endrin, and 0.21–8.81 ng/g-dw for DDT. It also showed that DDT, dieldrin and *β*-HCH were in abundance.

Based on the abovementioned results, the monitoring of the residues of OCPs in agricultural environment including soil, water, and sediment was completed in the three past decades. The residual levels of OCPs in the agricultural environment of Taiwan were in the range of traces to a few ppb, indicating that the relevant regulations for banning the use of OCPs have resulted in better environmental quality.

## 4. Regulatory System of Controlling Pesticides-POPs in Taiwan

In the past decades, the public concern about POPs in the environment has been rising and there is a push towards promulgating legislative frameworks for controlling/restricting their uses and distribution based on the environmental and health risks posed by these chemicals. Therefore, the central competent authorities began revising and strengthening existing laws and regulations, and even adding new management regulations for controlling pesticide-POPs in recent years, which were in accordance with the Stockholm Convention on POPs [13]. Table 4 shows the status of these pesticide-POPs with regards to the relevant laws/acts in Taiwan. Table 5 summarizes the environmental standards/limits of pesticide-POPs under the authorizations of the allied regulations, including the Water Pollution Control Act (WPCA), the Soil and Groundwater Pollution Remediation Act (SGPRA), the Drinking Water Management Act (DWMA), the Marine Pollution Control Act (MPCA), the Feeds Control Act (FCA), and the Labor Safety and Health Law (LSHL).

According to the information about the official websites at the central competent authorities of Taiwan, the following description is an overview on the major regulations concerning these pesticide-POPs.

### 4.1. Environmental Protection Administration (EPA)

#### 4.1.1. Toxic Chemical Substances Control Act (TCSCA)

The Toxic Chemical Substances Control Act (TCSCA), passed in Nov. 1986 and amended several times, was formulated to prevent toxic chemical substances from polluting the environment and endangering human health. As summarized in Table 6, some pesticides-POPs have been listed under the authorization of the TCSCA. Herein, the toxicity classification is subject to the following definitions:

Class-1 toxic chemical substance: chemical substances that are not prone to decompose in the environment and may pollute the environment or endanger human health due to bioaccumulation, bioconcentration or biotransformation.

Class-3 toxic chemical substances: chemical substances that endanger human health or the lives of biological organisms immediately upon exposure.

Table 6 also lists the announcements of their regulatory status, control level and the threshold regulation quantity. It should be noted that most pesticide-POPs listed in Table 6 have been designated as both the Class-1 and Class-3 toxic chemical substances due to their toxicological and persistent properties in the environment. This is consistent with incorporating them into the POPs under the Stockholm Convention.

#### 4.1.2. Water Pollution Control Act (WPCA)

This Act, passed in Jul. 1974 and amended several times, aims to control water pollution and ensure the cleanliness of water resources in order to maintain ecological systems, to improve the living environment and to promote public health. Under the authorization of the WPCA, the Taiwan EPA in consultation with the relevant industry competent authorities (*i.e.*, Ministry of Economic Affairs) thus promulgated the Effluent Standards. Because pesticides are highly toxic to aquatic organisms and may be discharged from agricultural activities and industrial effluents, some pesticide-POPs, including aldrin, DDT, dieldrin, endrin, heptachlor, lindane, and toxaphene, have been designated as target compounds in the Standards, as listed in Table 5.

#### 4.1.3. Soil and Groundwater Pollution Remediation Act (SGPRA)

This Act, passed in Feb. 1990 and recently amended in Feb. 2010, is formulated to prevent and remediate soil and groundwater pollution, to ensure the sustainable use of soil and groundwater, to improve the living environment, and to promote public health. Under the authorization of the SGPRA, the Taiwan EPA thus promulgated the Soil Pollution Control Standards and the Groundwater Pollution Control Standards, respectively. Because pesticides are likely to be occasionally released into the soil/water environment in agricultural applications and/or non-point sources, some pesticide-POPs, including aldrin, chlordane, DDT, dieldrin, endrin, heptachlor, and toxaphene, have been designated as chemical targets in the Standards, as listed in Table 5.

#### 4.1.4. Marine Pollution Control Act (MPCA)

This Act, passed in Nov. 2000, aims to control marine pollution, to protect the marine environment, to maintain the marine ecology, and to safeguard public health and sustainable use marine resources. Under the authorization of the MPCA, the Taiwan EPA thus promulgated the Standards for Marine Environment Categories and Marine Environment Quality based on the marine conditions. Because some pesticide-POPs, which are highly toxic to fish and other aquatic organisms, are likely to be released into the marine environment from agricultural discharges, they have been designated as target chemicals in the Standards, as listed in Table 5.

#### 4.1.5. Environmental Agents Control Act (EACA)

This Act, passed in Nov. 1997 and amended several times, is formulated to prevent harm from environmental agents, to maintain human health, and to protect the environment. Under the authorization of the MPCA, the Taiwan EPA announced the prohibited environmental agents, which contain components whose manufacture, processing, import, export, sale, or use have been officially banned. According to the related regulation promulgated by the Taiwan EPA, some pesticide-POPs, including aldrin, chlordane, DDT, dieldrin, heptachlor, hexachlorocyclohexane, and lindane, have been listed as the prohibited components, which are banned from being used as environmental agents, as listed in Table 5.

### 4.2. Department of Health (DOH)

Due to their lipophilic (hydrophobic) properties, POPs readily accumulate in human tissue following the ingestion of contaminated foods, and may induce the so-called food chain biomagnification, posing both carcinogenic and endocrine disrupting effects. The Food Sanitation Management Act (FSMA), initially passed in Jan. 1975 and amended several times, is enacted for the governing of food sanitation, safety and quality to protect the health of citizens. One of the main aims for the Act is to ensure the sanitation, safety and quality standards for foods, food cleansers, food utensils, food containers and food packaging being sold, which were prescribed by the central competent authority (*i.e.*, DOH). Under the authorization of the FSMA, the relevant regulation (“Residual Limits of Pesticides in Livestock and Poultry products”) pertains to the regulatory management of pesticide-POPs in the common foods (including meat, milk, and egg), as listed in Table 7. In the Regulation recently promulgated in Jun. 2008, the pesticide-POPs include aldrin, chlordane, DDT, dieldrin, endrin, lindane, and heptachlor.

### 4.3. Council of Agriculture (COA)

After the admission of Taiwan to the World Trade Organization (WTO) on January 1, 2002, the central competent authority, the Council of Agriculture (COA), has planned several policies and also revised/promulgated related regulations to promote the ecological operation by minimizing the use of pesticides and synthetic chemicals for the sustainable development of Taiwan’s agriculture. In this regard, there are two relevant laws pertaining to the regulatory management of POPs in the pesticides and feeds: the Pesticide Management Act (PMA) and the Feed Control Act (FCA). In the PMA, passed in Jan. 1972 and recently amended in Jul. 2007, the term “prohibited pesticides” denotes any and all agro-pesticides prohibited in public announcement by the central government from being manufactured, processed, repackaged, imported, exported, sold or used. Since the early 1970s, some pesticides, including aldrin, DDT, dieldrin, endrin, heptachlor, hexachlorocyclohexane, lindane and toxaphene, have been officially designated as prohibited ones, as listed in Table 3. The FCA, passed in Jan. 1973 and recently amended in Jan. 2002, was established to maintain the quality of feeds and to promote the development of the animal husbandry and the aquaculture industry so as to protect the public health. Under the authorization of the Article 20 in the FCA, the central government (*i.e.*, COA) thus promulgated the Pesticide Residue Limits in Feeds on 17 Jan. 2008. Some pesticide-POPs, including aldrin, DDT, dieldrin, heptachlor, and lindane, have been designated as chemical targets in the Limits, as listed in Table 5.

### 4.4. Council of Labor Affairs (CLA)

In 1980s, the Taiwanese government also undertook many regulatory practices that are pertinent to the improvement of the workplace environment. In this respect, the most important legislation regarding occupational environment is the Labor Safety and Health Law (LSHL). Under the authorization of the Act, it has carried out many enforcement rules or regulations for the purpose of preventing occupational accidents and protecting labor safety and health. Regarding the regulatory management of pesticide, some pesticide-POPs, including chlordane, heptachlor, and toxaphene, have been set the permissible exposure limit (PEL) to be 0.5 mg/m^3^ (8-h time-weighted average) with the “skin” notation (dermal absorption) in the Permissible Concentration Standards of Hazardous Substances in Occupational Workplace Air, as shown in Table 5.

## 5. Conclusions

The information about the environmental properties, use and toxicity (carcinogenity) of pesticide-POPs and their environmental levels in Taiwan has been extensively described and analyzed in the paper. Although the use of these chlorinated insecticides has been banned for agricultural use in Taiwan since the early 1970s, minor amounts of pesticide-POPs residues in soils and river sediments have still been detected. In order to control these chemicals in parallel with the international treaties, the Taiwan government has further adopted prevention policies and regulatory measures to enhance and review the control mechanisms for lowering the environmental and health risks of pesticide-POPs in recent years. Thereafter, the “National Implementation Plan of Republic of China (R.O.C., Taiwan) under the Stockholm Convention on Persistent Organic Pollutants” has been passed by the Cabinet in Jul. 2008, although Taiwan is currently not a party of the Convention. In this regard, the cross-ministerial joint-venture by the environment, health, agriculture, and labor authorities (*i.e.*, EPA, DOH, COA, and CLA) has worked together to develop legislative frameworks for pesticide-POPs. More importantly, it was found that the mean concentrations of pesticide-POPs and their metabolites have shown a significant decline in recent years, reflecting all the government and public efforts aim at eliminating their residues in the Taiwanese environment.

## Figures and Tables

**Table 1.** Chemical and physical information on pesticide-POPs a.

**Pesticide-POPs**	**CAS No.**	**Mol. formula**	**MW**	**Mpa(°C)**	**V**_**P**_**b(Pa)**	**Hc(Pa-m^3^/mol)**	**S**_**w**_**d(mg/L)**	***K***_***ow***_**e**
Aldrin	309-00-2	C_12_H_8_Cl_6_	364.9	104	0.005 (25 °C)	50.3 (25 °C)	0.02 (20 °C)	6.5
Chlordane (technical)	12789-03-6	C_10_H_6_Cl_8_	409.8	∼105	0.06 (25 °C)	4.9 (25 °C)	0.1 (25 °C)	5.5
Chlordecone	143-50-0	C_10_Cl_10_O	490.6	350	<0.03 (25 °C)	0.003 (25 °C)	7.6 (25 °C)	4.5
DDT	50-29-3	C_14_H_9_Cl_5_	354.5	108.5	3×10 ^−5^ (25 °C)	1.3 (25 °C)	0.0055 (25 °C)	6.2
Dieldrin	60-57-1	C_12_H_8_Cl_6_O	380.9	∼175	0.0005 (20 °C)	5.9 (25 °C)	0.17 (20 °C)	4.3
Endrine	72-20-8	C_12_H_8_Cl_6_O	380.9	∼228	0.0004 (20 °C)	0.8 (25 °C)	<10 ^−6^ (25 °C)	4.6
Heptachlor	76-44-8	C_10_H_5_Cl_7_	373.4	∼95	0.05 (25 °C)	150 (25 °C)	0.18 (20 °C)	5.3
Hexachlorobenzene	118-74-1	C_6_Cl_6_	284.8	228.8	0.0025 (20 °C)	35.1 (20 °C)	0.005 (25 °C)	5.5
α-Hexachlorocyclohexane	319-84-6	C_6_H_6_Cl_6_	290.8	158	0.006 (25 °C)	1.1 (25 °C)	2.0 (25 °C)	3.8
β-Hexachlorocyclohexane	319-85-7	C_6_H_6_Cl_6_	290.8	309	0.06 (25 °C)	0.07 (25 °C)	0.7 (20 °C)	3.8
Lindane	58-89-9	C_6_H_6_Cl_6_	290.8	113	0.007 (25 °C)	0.3 (25 °C)	2 (25 °C)	3.6
Mirex	2385-85-5	C_10_Cl_12_	545.5	485	0.0001 (20 °C)	43.3 (20 °C)	0.2 (24 °C)	5.3
Pentachlorobenzene	608-93-5	C_6_HCl_5_	250.3	86	1.0 (25 °C)	70 (25 °C)	0.56 (25 °C)	5.2
Toxaphene	8001-35-2	∼C_10_H_10_Cl_8_	413.8	65 90	0.0009 (25 °C)	0.6 (20 °C)	0.55 (20 °C)	4.8

a Melting point.

b Vapor pressure.

c Henry’s law constant.

d Solubility in water.

e Octanol/water partition coefficient (as logarithmic scale).

**Table 2.** Common uses of pesticide-POPs and their properties on endocrine disrupting and carcinogenity.

**Pesticide-POPs**	**Common uses**	**EDCsa**	**IARCbcarcinogenity**	**USNTPccarcinogenity**
Aldrin	Insecticide	Listed	Group 3	-- d
Chlordane	Insecticide	Listed	Group 2B	--
Chlordecone	Insecticide	Listed	Group 2B	Reasonable suspected
DDT	Insecticide	Listed	Group 2B	Reasonable suspected
Dieldrin	Insecticide	Listed	Group 3	--
Endrin	Insecticide	Listed	Group 3	--
Heptachlor	Insecticide	Listed	Group 2B	--
Hexachlorobenzene	Bactericide	Listed	Group 2B	Reasonable suspected
α-Hexachlorocyclohexane	Insecticide	Listed	Group 2B	Reasonable suspected
β-Hexachlorocyclohexane	Insecticide	Listed	Group 2B	Reasonable suspected
Lindane	Insecticide	Listed	Group 2B	Reasonable suspected
Mirex	Insecticide	Listed	Group 2B	Reasonable suspected
Pentachlorobenzene	Fungicide intermediate, fire retardant	--	--	--
Toxaphene	Insecticide	Listed	Group 2B	Reasonable suspected

a Listed in the endocrine disrupting chemicals (EDCs) by the Ministry of the Environment (Japan).

b Overall evaluations of carcinogenicity to humans by the International Agency for Research on Cancer (IARC); Group 1: Carcinogenic to humans; Group 2A: Probably carcinogenic to humans; Group 2B: Possibly carcinogenic to humans; Group 3: Not classifiable as to carcinogenicity to humans.

c United States National Toxicology Program (USNTP) by the Department of Health and Human Services; Reasonable suspected: Reasonably anticipated to be a human carcinogen.

d Not listed.

**Table 3.** The data list of pesticide-POPs officially banned or restricted in Taiwan.

**Pesticide-POPs**	**Banned as pesticidesa**	**Banned as toxic chemicalsb**	**Banned as environmental agentsc**	**Comments**
Aldrin	Jan. 1, 1975	May 2, 1989	Apr. 20, 1998	
Chlordane	--	Jun. 24, 1988	Apr. 20, 1998	Never registered
Chlordecone	--	--	--	Never registered
DDT	Jul. 1, 1973	May 2, 1989	Apr. 20, 1998	
Dieldrin	Jan. 1, 1975	May 2, 1989	Apr. 20, 1998	
Endrin	Jan. 1, 1971	May 2, 1989	--	
Heptachlor	Jan. 1, 1975	May 2, 1989	Apr. 20, 1998	
Hexachlorobenzene	--	Dec. 24, 1993	--	Never registered
α-Hexachlorocyclohexane	Jan. 1, 1975	May 2, 1989	Apr. 20, 1998	
β-Hexachlorocyclohexane	Jan. 1, 1975	May 2, 1989	Apr. 20, 1998	
Lindane	Aug. 7, 1984	May 2, 1989	Apr. 20, 1998	
Mirex	--	--	--	Never registered
Pentachlorobenzene	--	--	--	Never registered
Toxaphene	Jul. 19, 1983	May 2, 1989	--	

a Under the authorization of the Pesticide Management Act (Council of Agriculture).

b Under the authorization of the Toxic Chemical Substances Control Act (Environmental Protection Administration).

c Under the authorization of the Environmental Agents Control Act (Environmental Protection Administration).

**Table 4.** Control/restriction regulations governing persistent organic pollutants (POPs) in Taiwan a.

**POPs**	**EPA**						**COA**		**DOH**	**CLA**
	**DWMA**	**EACA**	**MPCA**	**SGPRA**	**TCSCA**	**WPCA**	**PMA**	**FCA**	**FSMA**	**LSHL**
Aldrin		•	•	•	•	•	•	•	•	•
Chlordane		•		•	•			•	•	•
Chlordecone										
DDT		•	•	•	•	•	•		•	
Dieldrin		•	•	•	•	•	•	•	•	•
Endrin			•	•	•	•	•		•	•
Heptachlor		•	•	•	•	•	•	•	•	•
α-Hexachlorocyclohexane		•			•		•	•	•	
β-Hexachlorocyclohexane		•			•		•	•	•	
Lindane	•	•	•		•	•	•	•	•	•
Mirex										
Pentachlorobenzene										
Toxaphene			•	•	•	•	•		•	•

a Central competent authorities of Taiwan, given below

EPA: Environmental Protection Administration; COA: Council of Agriculture; DOH: Department of Health; CLA: Council of Labor Affairs.

Central Law and Act of Taiwan, given below

DWMA: Drinking Water Management Act; EACA: Environmental Agents Control Act; FCA: Feed Control Act; FSMA: Food Sanitation Management Act; LSHL: Labor Safety and Health Law; MPCA: Marine Pollution Control Act; PMA: Pesticide Management Act; SGPRA: Soil and Groundwater Pollution Remediation Act; TCSCA: Toxic Chemical Substances Control Act; WPCA: Pollution Control Act.

**Table 5.** Standards/limits of pesticide-POPs under the promulgation by the central competent authorities (*i.e.*, EPA, COA and CLA) of Taiwan.

**Act/Law**	**Regulation (unit)**	**Standard/limit**							
		**Aldrin**	**Chlordane**	**DDT**	**Dieldrin**	**Endrin**	**Heptachlor**	**Lindane**	**Toxaphene**
WPCA	Effluent Standards (mg/L)	0.003		0.001	0.003	0.0002	0.001	0.004	0.005
SGPRA	Standards for Soil Pollution Control (mg/kg)	0.04	0.5	3	0.04	20	0.2		0.6
	Standards for Groundwater Pollution Control (mg/L)		0.002/0.02 a						0.003/0.03
DWMA	Standards for Drinking Water Quality (mg/L)							0.0002	
	Standards for Drinking Water Source Quality (mg/L)							0.004	
MPCA	Standards for Marine Environment Categories and Marine Environment Quality (mg/L)	0.003		0.001	0.003	0.002	0.001	0.004	0.005
FCA	Pesticide Residue Limits in Feeds (ppm)	0.02		0.5	0.02		0.02	0.05	
LSHL	Permissible Concentration Standards of Hazardous Substances in Occupational Workplace Air (mg/m^3^)	(skin) b	0.5 (skin)		(skin)	(skin)	0.5 (skin)	(skin)	0.5 (skin)

a The former (*i.e.*, 0.002 mg/L) is the control standard for the groundwater within the protection zone of drinking water sources. The latter (*i.e.*, 0.02 mg/L) is the control standard for the groundwater sources except for the protection zone of drinking water sources.

b The “skin” notation indicates that the contact with skin, eyes, and mucous membranes can contribute to the overall exposure.

**Table 6.** Pesticide-POPs designated as toxic substances under the Toxic Chemical Substances Control Act in Taiwan.

**Pesticide-POPs**	**Control level (wt%)**	**Threshold Regulation quantity (kg)**	**Toxicity classification**	**Regulatory status**
Aldrin	1	50	1, 3	Manufacture, import, sale, and use prohibited a
Chlordane	1	50	1, 3	Manufacture, import, sale, and use prohibited
DDT	1	50	1, 3	Manufacture, import, sale, and use prohibited
Dieldrin	1	50	1, 3	Manufacture, import, sale, and use prohibited
Endrin	1	50	1, 3	Manufacture, import, sale, and use prohibited
Heptachlor	1	50	1, 3	Manufacture, import, sale, and use prohibited
Hexachlorobenzene	1	50	1	Manufacture, import, sale, and use prohibited
α-Hexachlorocyclohexane	1	50	1, 3	Manufacture, import, sale, and use prohibited
β-Hexachlorocyclohexane	1	50	1, 3	Manufacture, import, sale, and use prohibited
Lindane	1	50	1, 3	Manufacture, import, sale, and use prohibited
Toxaphene	1	50	1	Manufacture, import, sale, and use prohibited

a It is still used for the following purposes: testing, research and education.

**Table 7.** Residue limits of pesticide-POPs in livestock and poultry products under the promulgation by the central competent authority (*i.e.*, DOH) of Taiwan.

**Pesticide-POPs**	**Residual location**	**Category**	**Residual limit (ppm)**	**Comment**
Aldrin	Meat	Livestock	0.2	Based on fat
	Milk		0.006	
	Egg		0.1	

Chlordane	Meat	Livestock	0.05	Based on fat
	Milk		0.002	
	Meat	Poultry	0.5	Based on fat
	Egg		0.02	

DDT	Meat	Livestock	5	Based on fat
	Milk		0.05	
	Egg		0.5	

Dieldrin	Meat	Livestock	0.2	Based on fat
	Milk		0.006	
	Egg		0.1	

Endrin	Meat	Livestock	0.1	Based on fat
	Milk		0.0008	
	Meat	Poultry	1	Based on fat
	Egg		0.2	

Lindane	Meat	Cattle/Pig/Sheep	2	Based on fat
	Milk		0.01	
	Meat	Poultry	0.7	Based on fat
	Egg		0.1	

Heptachlor	Meat	Livestock/Poultry	0.2	Based on fat
	Milk		0.006	
	Egg		0.05	
